# Circulating miRNAs as Potential Biomarkers for Patient Stratification in Bipolar Disorder: A Combined Review and Data Mining Approach

**DOI:** 10.3390/genes13061038

**Published:** 2022-06-10

**Authors:** Alexandra R. Clausen, Simon Durand, Rasmus L. Petersen, Nicklas H. Staunstrup, Per Qvist

**Affiliations:** 1 Department of Biomedicine, Aarhus University, 8000 Aarhus, Denmark; 201409315@post.au.dk (A.R.C.); simon.r.durand@gmail.com (S.D.); rasmuslp37@hotmail.com (R.L.P.); nhs@clin.au.dk (N.H.S.); 2 iPSYCH, The Lundbeck Foundation Initiative for Integrative Psychiatric Research, 8000 Aarhus, Denmark; 3 Centre for Integrative Sequencing, iSEQ, Aarhus University, 8000 Aarhus, Denmark; 4 Centre for Genomics and Personalized Medicine, CGPM, Aarhus University, 8000 Aarhus, Denmark; 5 Blood Bank and Immunology, Aarhus University Hospital, 8200 Aarhus, Denmark; 6 Department of Health Science and Technology, Aalborg University, 9200 Aalborg, Denmark

**Keywords:** miRNA, bipolar disorder, epigenetics, biomarkers, exposome, pharmacological biomarkers

## Abstract

Bipolar disorder is a debilitating psychiatric condition that is shaped in a concerted interplay between hereditary and triggering risk factors. Profound depression and mania define the disorder, but high clinical heterogeneity among patients complicates diagnosis as well as pharmacological intervention. Identification of peripheral biomarkers that capture the genomic response to the exposome may thus progress the development of personalized treatment. MicroRNAs (miRNAs) play a prominent role in of post-transcriptional gene regulation in the context of brain development and mental health. They are coordinately modulated by multifarious effectors, and alteration in their expression profile has been reported in a variety of psychiatric conditions. Intriguingly, miRNAs can be released from CNS cells and enter circulatory bio-fluids where they remain remarkably stable. Hence, peripheral circulatory miRNAs may act as bio-indicators for the combination of genetic risk, environmental exposure, and/or treatment response. Here we provide a comprehensive literature search and data mining approach that summarize current experimental evidence supporting the applicability of miRNAs for patient stratification in bipolar disorder.

## 1. Introduction

Bipolar disorder (BD) is a common, chronic illness characterized by extreme mood swings, high rates of morbidity, suicide, and an overall lowered quality of life [1,2]. The diagnosis of BD is based on clinical interviews and observations, and the current Diagnostic and Statistical Manual of Mental Disorders (DSM-V) classifies BD into categorical subtypes based on the intensity of manic episodes (Type I and II) [2]. The clinical trajectory and manifestation of BD is, however, heterogeneous and include overlaps with schizophrenia (SZ) and other affective disorders [1,2,3,4]. Consequently, up to 60% of patients are initially misdiagnosed with unipolar depressive disorder (MDD) and patients experience a mean delay of 5–10 years from symptom onset to BD diagnosis [5,6].

The etiology of BD is multifactorial, with genetic and environmental factors (EFs) contributing to abnormal brain development and disease progression individually [7,8,9,10,11], and through complex gene–environment interactions (GxE) [12]. The genetic component is substantial with an estimated heritability of 60–85% based on family and twin studies [13,14,15]. Robust association of more than 60 loci has been identified in the, to date, largest BD genome-wide associations study (GWAS) [11]. In support of the diagnostic sub-classification of BD, the genetic correlation between BD type I and II is high, but incomplete [8]. Known EFs associated with BD can be grouped into three categories based on developmental timing; prenatal (e.g., infection during pregnancy, Vitamin D levels); childhood (e.g., maltreatment, parental loss), and adolescence/adulthood (e.g., cannabis and stressful life events) [12,16,17]. While GxE have been investigated on a genome-wide scale in genome-wide gene–environment interaction studies for a number of psychiatric phenotypes [18,19,20,21,22,23], studies of GxE in BD have, with a few exceptions, been limited to candidate gene approaches (e.g.: variation in *BDNF*, stressful life event, and worst episodes of depression and mania [24], childhood trauma and age of BD onset, severity, and chronicity [25]) and a handful of other GxE associations [26,27,28,29,30,31]—recently reviewed by Misiak et al. [32] and Musci et al. [33]). In addition, pharmacogenomic studies have identified associations between genetic variation and drug response/adverse effects in GWASs and in targeted candidate studies of genes implicated with drug metabolism and pharmacodynamics (recently reviewed by Cuéllar-Barboza et al. [34]). Importantly, however, neither the identified environmental or genetic risks are unique to BD, but generally shared between psychiatric diagnostic entities [12,35,36,37]. Collectively, this suggests that psychiatric phenotypes are shaped in a concerted interplay between common set of endo- and exogeneous risks [12,17,36]. Identification of peripheral biomarkers that capture the combined risk burden may thus significantly improve the diagnosis, prognosis, and treatment of BD.

**Figure 1:**
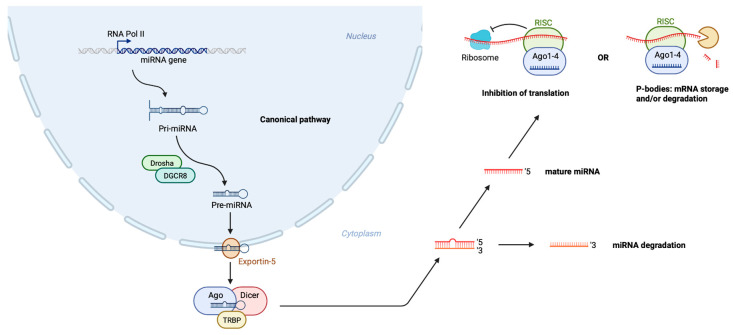
Schematic representation of miRNA biogenesis and mechanism of action. RNA polymerase II transcribes miRNA genes into primary miRNA (pri-miRNA) in the cellular nucleus. The enzyme, Drosha process pri-miRNA into precursor miRNA (pre-miRNA), which is exported out to the cytoplasm by exportin 5. In the cytoplasm the pre-miRNA is processed into a double-stranded miRNA/miRNA*duplex by Dicer. Two mature miRNA species may be generated from the 5′ and 3′ arms of the duplex (referred to as 5p and 3p), however in most cases one is degraded and the other dominates. The mature miRNA is incorporated into RISC (RNA inducing silencing complex). The RISC with bound miRNA, then binds to target mRNA, triggering degradation or inhibition of translation.

Changes in epigenetic marks bridge environmental exposures and gene regulation, and may thus serve as a measurable gauge of environmental influence on disease risk [38,39]. These include, among other things, DNA methylation, covalent histone modifications, and noncoding RNAs. Particularly, microRNAs (miRNAs), which expression and function is influenced by both genetic risk variants [40,41] and the risk exposome [42,43,44], have been extensively studied in the context of psychiatric disorders [45,46,47,48]. miRNAs act as important regulators of post-transcriptional gene expression through sequence-specific binding to target mRNAs [49], thereby impacting on their translation and/or stability (Figure 1). A single mRNA may be regulated by multiple miRNAs [50], and, conversely, some miRNAs have the potential to target hundreds of mRNAs [51]. Although only few miRNAs are expressed in a brain-specific or enhanced way, more than 70% of all transcribed miRNAs are found in the CNS [52,53]. Within the brain, their expression often differs between brain tissues and/or across developmental stages [54,55,56]. Intriguingly, miRNAs can be released from CNS cells and enter into circulatory bio-fluids where they remain remarkably stable [57]. Alterations in the abundance of miRNAs has been documented in brain tissue as well as in blood from patients with psychiatric disorders [58]. It is thus possible that peripheral circulatory miRNAs may act as bio-indicators for the combination of genetic and environmental risk exposure as well as treatment response in BD [59].

In this combined data and literature review, we assess the genomic evidence that support peripheral circulatory miRNAs as potential biomarkers for patient stratification in BD.

## 2. Materials and Methods

### 2.1. Literature Review

Two independent comprehensive literature searches were performed on the electronic databases Embase (http://www.embase.com, accessed on 23 May 2022) and PubMed (http://www.ncbi.nlm.nih.gov/pubmed, accessed on 23 May 2022). Abstracts and titles were screened using eligibility criteria based on the PICOS (Population, Intervention, Comparison, Outcomes, and Study) framework and according to the PRISMA (Transparent Reporting of Systematic Reviews and Meta-analyses) guidelines [60]. Reference lists were manually evaluated and studies assessing less than six miRNAs as well as unpublished articles; meta-analyses; abstracts and retrospective studies were excluded.

(1) Search on PubMed was performed with string 1: (‘bipolar disorder’[MeSH]) AND (‘microRNA’[MeSH]). Whereas on EMBASE, string 2: (‘bipolar disorder’[Broad Search]) AND (‘microRNA’[Emtree]) AND (´biological marker´[Broad Search]) was combined with string 3: (‘bipolar disorder’[Broad Search]) AND (‘microRNA’[Emtree]) AND (‘blood’[Broad Search]).

Filters applied on PubMed were Age: adult (18–44), middle age (44–65) and Species: human. On EMBASE these were Age: adult (18–64) and Study type: human. Only original studies investigating miRNA abundance in peripheral blood were included. Participants had to be diagnosed with BD type I or II and compared to unaffected family members and/or unrelated, healthy controls. Cyclothymia (BD type III) was not included. This search resulted in 144 studies after removing duplicates and 58 after application of filters. All reviews and preliminary papers were excluded, leaving 52 studies. The 52 papers were assessed according to the inclusion criteria, and four articles were deemed eligible. These were quality assessed using STROBE statements leading to one study being excluded due to poor reporting of research quality [61].

(2) The following search strings were applied and combined using PubMed, string 1: (‘bipolar disorder’[MeSH]) AND (‘drug’[All Fields]) AND (‘efficacy’[All Fields]); string 2: (‘bipolar disorder’[MeSH]) AND (‘micrornas’[MeSH] AND (‘antipsychotic agents’[All Fields]); string 3: (‘bipolar disorder’[MeSH Terms]) AND (‘micrornas’[MeSH Terms]) AND (‘lithium’[All Fields]). Using EMBASE the following strings were applied in combination string 4: (‘bipolar disorder’[Broad Search]) AND (‘microRNA’[Emtree]) AND (‘drug efficacy’[Broad Search]); string 5: (‘bipolar disorder’[Broad Search]) AND (‘microRNA’[Emtree]) AND (‘antipsychotic agents’[Broad Search]).

Afterwards, the filters Species: ‘human’ and Pub. type: ‘article’ were applied on the search results. Only participants diagnosed with BD type I or II were included, and cell lines derived from blood samples from patients were accepted. Comparisons comprised responders versus non-responders, as well as, medicated versus un-medicated.

Combining the search outputs from PubMed and EMBASE resulted in 41 original studies after removing duplicates (Figure S2). Applying the filters reduced the number to 14 articles, which were finally assessed based on the inclusion criteria. Three articles were deemed eligible and included in the review.

### 2.2. Data Review

GWAS data: Summary statistics from the, to date, largest GWAS study on BD [11], was downloaded from the Psychiatric Genomics Consortium (PGC) homepage. Genomic boundaries of genome-wide associated loci, as defined by independent significant SNPs (r^2^ ≤ 0.6), were identified using FUMA [62]. A list of known miRNAs and their genomic positions was extracted from the Biomart community portal hosted by Ensembl [63,64]. miRNAs located in genome-wide associsated loci were identified using Galaxy, an open source, web-based platform for data intensive biomedical research [65]. Regional association plots of loci harbouring selected miRNAs were generated with LocusZoom.js [66].

eQTL analysis: cis-eQTL mapping of BD risk-increasing genetic variants in the assessed BD GWAS [11] was performed using FUMA based on GTEX v8 (Blood and Brain tissues only) [67] with a SNP-Gene significance threshold of FDR < 0.05. Further, the GTExportal based on GTEX v8 (significant single-tissue in all tissues) [67].

Bulk transcriptomic data: Normalized expression values (Reads Per Kilobase of transcript, per Million mapped reads, RPKM) for selected miRNAs for 16 different brain tissues in the developing and mature brain was downloaded from www.brainspan.org, (accessed on 17 December 2021) [68]. Developmental stages were defined as: Early fetal 8 ≤ Age < 13 postconceptional week (PCW); Early mid fetal 13 ≤ Age < 19 PCW; Late mid fetal 19 ≤ Age < 24 PCW; Late fetal 24 ≤ Age < 38 PCW; Neonatal 0 ≤ Age < 6 Postnatal months (M); Late infancy 6 M ≤ Age ≤ 1 Year (Y); Early childhood 1 < Age < 6 y Middle and late childhood 6 ≤ Age < 12 y; Adolescence 12 ≤ Age < 20 y; Young adulthood 20 ≤ Age < 40 y. Average RPKM within developmental stages were plotted.

Expression characteristics of specifically 3p and 5p strands in human cortical tissue was assessed for selected miRNAs in sequencing data reported by Hoss et al. [69]. Data was downloaded from NCBI’s Gene Expression Omnibus (GEO) [70] through GEO Series accession number GSE64977.

Single-cell transcriptomic data: Cell-type-specific expression characteristics of selected miRNAs was assessed in data from 49,495 nuclei from multiple human cortical tissues sampled from postmortem and neurosurgical brains from adult (18–68 years) donors [71]. Normalized expression values (Counts per million, CPM) calculated for each nuclei, was plotted against annotated cell types in the atlas using the Allen Brain Map Transcriptomic explorer [72].

miRNA target prediction: Target prediction was performed for selected miRNAs (3p and 5p) using DIANA-microT-CDS [73], with a prediction score threshold filter of 0.7.

Gene set association analysis: Gene set analysis was performed with MAGMA [74] using default settings, based on summary statistics from the assessed BD GWAS [11]. SNPs within the HLA locus and imputed SNPs with info score < 0.8, were excluded from the analyses.

Gene set enrichment analysis: Gene set enrichment analyses were performed with FUMA [62] with all genes as background reference list, excluding the HLA locus. A significance threshold was set at FDR of *p*-value < 0.05.

## 3. Results

### 3.1. Literature Reviews

#### 3.1.1. Literature Review of Changes in Peripheral Circulating miRNAs in Bipolar Disorder

The included studies were all cross-sectional (Figure S1). Three studies compared blood miRNA profiles between medicated BD type I and healthy controls, whereas one assessed miRNA in blood from BD type II medication-free patients to healthy controls. Methodological variation between studies included study material (exosomal/extra- and intracellular miRNA), ethnicity, and medication status of patients, disorder stage, diagnostic instrument, and miRNA quantification methods (Quantitative Real time-PCR (qRT-PCR) and microarray).

##### BD Type I

The study by Camkurt et al. compared the abundancy of peripheral miRNAs in patients with BD type I in euthymic and manic stage to healthy controls [75]. The miRNAs investigated were preselected based on data from epigenetic studies of psychiatric disorders and comprised: *miR-26b-5p*; *miR-9-5p*; *miR-29a-3p*; *miR-106a-5p*; *miR-106b-5p*; *miR-107*; *miR-125a-3p*; and *miR-125b-5p* [76,77,78,79,80,81,82].

Overall, assessed miRNAs were more abundant in BD subjects compared to controls, with more pronounced differences observed in the group of manic patients than in the euthymic patient group. In the euthymic group alone, only *miR-107* and *miR-125a-3p* were significantly upregulated after correction for multiple testing. In the group of manic patients all of the investigated miRNAs, except for *miR-125a-3p,* were significantly upregulated. When the euthymic and manic group were combined *miR-29a-3p, miR-106b-5p, miR-107* and *miR-125a-3p* were found upregulated (Table 1).

Fries et al. detected a total of 380 miRNAs in peripheral exosomes, of which 33 showed nominal significant differences in abundancy between the BD subjects and controls. However, none of these remained after correction for multiple testing [83].

Ceylan et al. compared the abundance of exosomal miRNA in BD subjects in the manic, euthymic or depressed stage to healthy controls [84]. Of the 752 exosomal miRNAs identified, five were found upregulated and eight downregulated, when comparing the combined BD group to controls. However only the upregulation of *miR-185-5p* and the downregulation of *miR-484*, *miR-652-3p*, and *miR-142-3p* remained significant following correction for multiple testing (Table 1). Notably, dysregulation of the combined set of miRNAs was found to show a specificity of 75% and a sensitivity of 87% to the BD diagnosis.

##### BD Type II

The study by Lee et al. determined candidate miRNAs by using next generation sequencing on six blood samples (three BD and three healthy controls) identifying > 280 miRNAs [59]. Twenty-four miRNAs were found significantly upregulated and five downregulated. Of these, the most statistically significant differentially regulated miRNAs (*miR-7-5p*, *miR-23b-3p*, *miR-142-3p*, *miR-221-5p*, *miR-370-3p* and *miR-145-5p*) were further explored in peripheral blood samples from 79 BD subjects and 95 controls. Here, *miR-7-5p*, *miR-23b-3p*, *miR-142-3p*, *miR-221-5p*, and *miR-370-3p* was again found upregulated in BD patients compared to healthy controls (Table 1). A testing cohort was afterwards used to evaluate the diagnostic efficacy of the four miRNAs, and a specificity of 90% and sensitivity of 85% was reported.

#### 3.1.2. Literature Review of Exposome-Associated Changes in Peripheral Circulatory miRNAs in Bipolar Disorder

Next, we conducted a literature search for documented changes in peripheral circulatory miRNA abundancy in BD patients associated with exposure to the BD-associated exposome (Figure S2). Whereas no studies have examined changes to the miRNA profile in patients exposed to classical BD-associated environmental risk factors, a systematic search for studies assessing changes in peripheral circulatory miRNAs associated with treatment in BD patients identified three original research papers.

Chen et al. analyzed the expression of 13 preselected miRNAs in 20 BD type I-derived lymphoblastoid cell lines with or without lithium treatment in culture [85]. The authors reported seven miRNAs that displayed nominal significant changes in abundancy between treated and non-treated cells. Of these, four remained significant after correcting for multiple testing (Table 2).

Lim et al. examined changes in miRNA expression in 10 BD type I patients before and after 12 weeks of treatment with the antipsychotics, Asenapine or Risperidone [86]. They identified 16 miRNAs that were differentially expressed after treatment with Asenapine in comparison to the control group. In the Risperidone-administered group, three miRNAs were downregulated compared to the control group (Table 2).

Pisanu et al. measured genome-wide miRNA expression in lymphoblastoid cell lines from 24 BD patients [87]. Patients were categorized as excellent responders (*n* = 12, score ≥ 7) and non-responders (*n* = 12, score ≤ 2) to lithium according to the “Retrospective Criteria of Long-Term Treatment Response in Research Subjects with Bipolar Disorder” (Alda scale) [88]. The authors reported 52 miRNAs that differed significantly in abundancy between lithium excellent responders and non-responders (Table 2).

### 3.2. Data Mining

#### 3.2.1. Genetic Risk in miRNA-Hosting Genomic Loci in Bipolar Disorder

**Figure 2:**
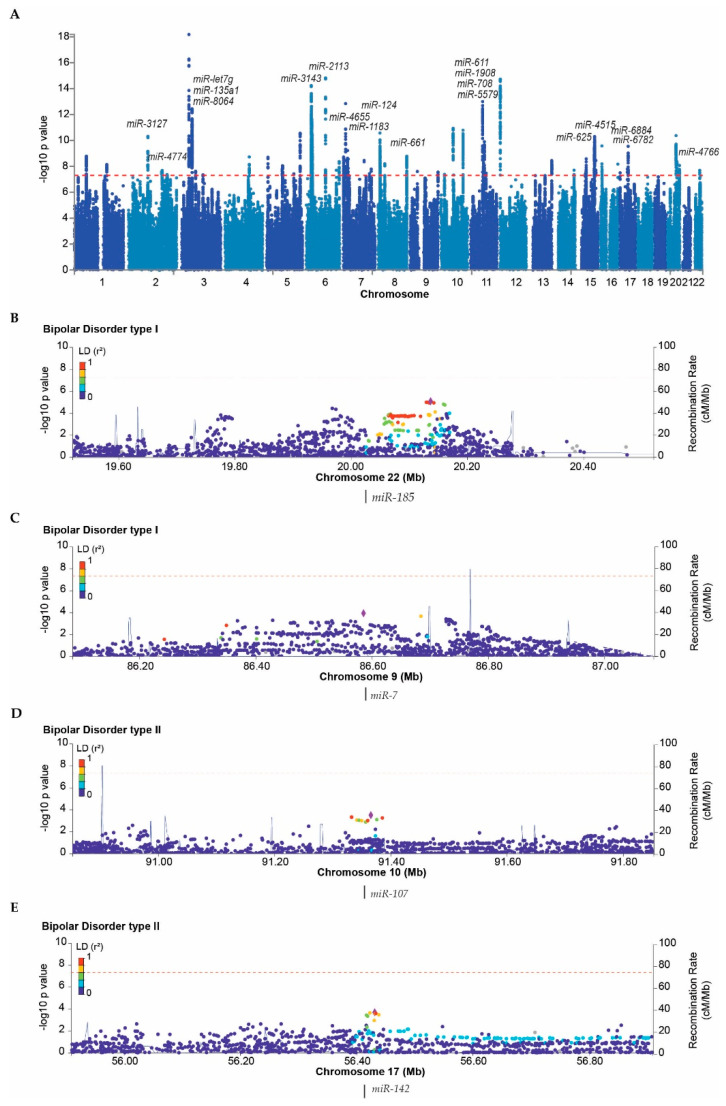
Genetic association to BD in miRNA-harboring loci. (**A**) Manhattan plot showing 64 genome-wide significant (GWS) loci identified in the, to date, largest GWAS on BD samples. miRNA-encoding genes located within each GWS locus are listed above loci. (**B**,**C**) Regional association plots for BD type I: (**B**) *miR-185* locus (**C**) *miR-7* locus, and (**D**,**E**), and for BD type II: (**D**) *miR-107* locus and (**E**) *miR-142* locus. The purple diamond indicates the most significant single-nucleotide polymorphism (SNP) of each region, and nearby SNPs are color-coded to show their LD relationships with the top SNP (r^2^ < 0.2, dark blue; 0.2 ≤ r^2^ < 0.4, light blue; 0.4 ≤ r^2^ < 0.8, green; r^2^ > 0.8, red) based on Hapmap3 EUR. Dotted line marks GWS cutoff.

The tissue abundance of miRNAs is strictly controlled at the transcriptional and posttranscriptional level by molecular mechanisms that are influenced by both genetic and environmental factors [89]. Genetic variants may directly affect gene transcription, miRNA processing or modulate the genomic response to environmental stimuli [90], and environmental factors may regulate transcriptional activity or affect miRNA turnover [91]. We assessed if the transcription of the reported dysregulated circulatory miRNAs occurs from genomic loci associated with BD. For this study we utilized summary statistics from the, to date, largest BD genome-wide association study (GWAS) meta-analysis of BD type I and II [11], comprising 41,917 BD cases and 371,549 healthy controls of European ancestry. We found that 17 of the 64 identified genome-wide significant (GWS) loci harbor genes encoding miRNAs, with several harboring more than one (Figure 2A and Table S1). Among these, multiple miRNAs have documented roles in neuronal functioning [92], and/or behavior [93]. However, none of the miRNAs located in GWS loci were identical to the miRNAs dysregulated in blood of BD patients. The majority of associated signals in GWASs, however, map to non-coding sequences, and the functional impact of the associated genetic variants may not lie within the associated loci. Hence, we further assessed if any of the risk increasing variants in GWS loci are associated with changes in miRNA expression (expression quantitative loci (eQTLs)) in blood and brain tissues. None of the associated variants were cis-eQTLs for miRNA-encoding genes. Conversely, we investigated if any of the reported differentially abundant miRNAs in BD patients had known eQTLs. Expect for *miR-221* with a single known eQTL (rs138686331) in skin, none had any known eQTLs.

The top associated loci, however, only account for a fraction of the estimated heritability of BD. Hence, we looked at regional genetic association in loci hosting the BD type I and II circulatory miRNAs in question. As expected, no loci hosted signals that surpassed the GWS cutoff, but several sets of markers near (<0.1 MB) or within LD blocks spanning the assessed miRNAs, reached a nominal significance of *p* < 0.0001 for each or both of the BD subtypes (Figure 2B–E, Figures S3 and S4). For BD type I, this included *miR-185* and *miR-7* (Figure 2B,C), whereas in BD type II, *miR-107* and *miR-142* showed nominal significant association at the defined threshold (Figure 2D,E). Only SNPs in the *miR-484* locus showed nominal significant association to both BD subtypes (Figures S3G and S4G).

#### 3.2.2. Expression Characteristics of Putative Bipolar Disorder miRNA Biomarkers in the Human Brain

Our combined literature and data review identified five miRNAs (*miR-142*, *miR-106b*, *miR-652*, *miR-125a*, and *miR-221*) that were both differentially abundant in peripheral blood from BD patients compared to healthy controls, and differentially expressed following treatment with antidepressants/mood stabilizers or between treatment responders and non-responders. In order to assess their putative role in the pathobiology of BD, we examined their expression characteristics in the developing and mature human brain using public deposited data [68,69,71].

##### miR-106b

**Figure 3:**
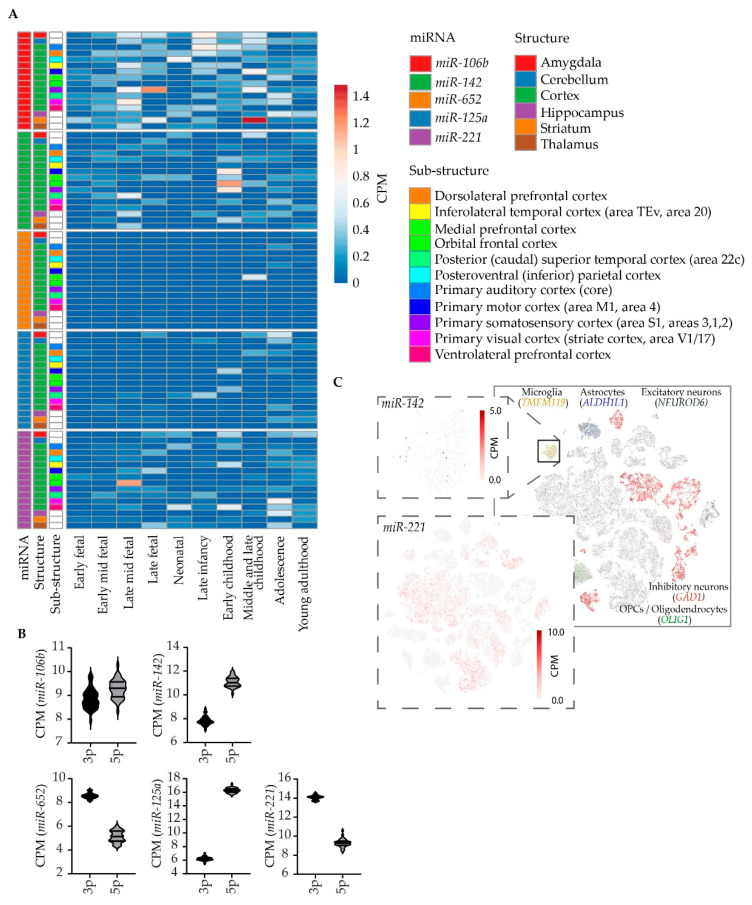
Expression characteristics of candidate miRNAs in the postmortem human brain and single nuclei derived from human brain tissue. (**A**) Plotted are the normalized expression values (RPKM) of putative miRNA biomarkers in multiple human brain tissues sampled throughout development. (**B**) Plotted are normalized expression (CPM) of single nuclei candidate miRNA strand-specific counts (5p and 3p) in adult human cortex. (**C**) Shown are normalized expression values for miR-142 and miR-221 in single cell sequencing data from human cortical tissue. For reference, markers of major brain cell types are shown.

Of the five examined miRNAs, *miR-106b* was the most abundantly expressed in brain tissue, peaking in striatal tissue in childhood (Figure 3A). Expression is here measured collectively for 3p and 5p strands. Whereas one miRNA strand is typically degraded, co-existence of miRNA-5p and -3p species is increasingly being reported [94]. In a study, particularly measuring the 3p and 5p miRNA strands in cortical tissue from adult human donors [69], *miRNA-106b-3p* and *-5p* were almost equally abundant (Figure 3B). In one of the most comprehensive efforts to map gene expression in single nuclei sampled from cortical tissue from adult human donors [71], no brain cell-type specific expression was seen for *miR-106b*.

##### miR-142

*miR-142* expression peaked in cortex in early childhood, with particularly high abundance in the orbital frontal cortex (Figure 3A). Although *miRNA-142-5p* is the dominant strand in the adult human cortex, both strands are abundant (Figure 3B). Intriguingly, in brain-derived cells, *miR-142* was nearly exclusively detected in microglia—albeit only in a minor fraction of glial nuclei at the applied sequencing depth (Figure 3C).

##### miR-652

*miR-652* was only detected in substantial abundancy in medial frontal cortex from donors in their middle- and late childhood in the brainspan dataset (Figure 3A). The 3p strand was two-fold more abundant than the 5p strand in cortex (Figure 3B) and *miR-652* expression was restricted to a very small subset of nuclei and showed no specificity to any cell type in the single cell expression dataset.

##### miR-125a

*miR-125a* was detected in all cell types in the adult cortex but displayed overall low expression in brain tissue—peaking in amygdaloid tissue in adolescence (Figure 3A). The 5p strand was significantly more abundant than 3p in the adult cortex (Figure 3B).

##### miR-221

*miR-221* peaked in the orbital frontal cortex in the mid-late fetal stage but displayed an overall increase in abundance towards adolescence and adulthood in both cortical and sub-cortical tissues (Figure 3A). 3p was the dominant strand in the adult cortex (Figure 3B). Interestingly, *miR-221* was exclusively detected in nuclei expressing markers of excitatory neurons (Figure 3C).

#### 3.2.3. Predicted Regulomes of Putative miRNA Biomarkers and Their Implication in Mental Health

**Figure 4:**
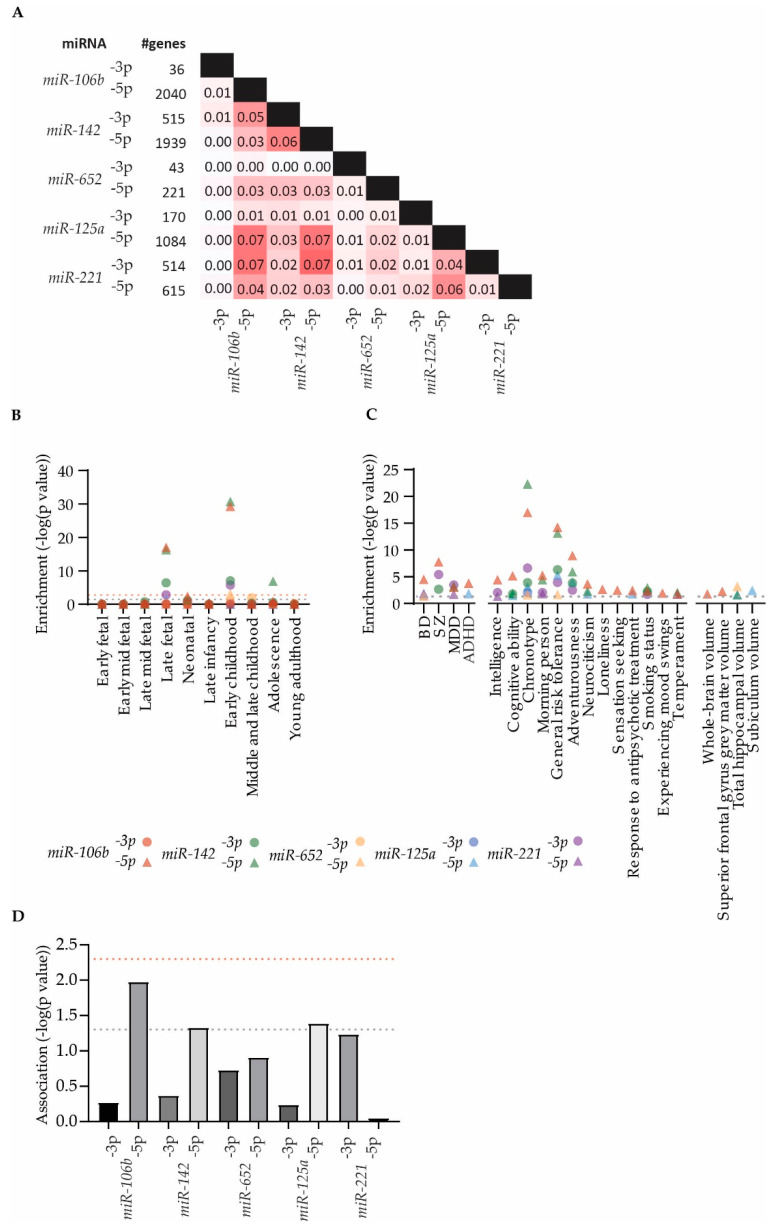
Characterization of predicted miRNA target genes. (**A**) Jaccard similarity index of predicted target gene sets of the five putative BD miRNA biomarkers. (**B**) Enrichment of genes with differential expression in brain tissue at 10 developmental stages. Grey dotted line marks nominal significant cutoff at *p* = 0.05, while red dotted line marks significance cutoff adjusted for multiple testing. (**C**) Enrichment of GWAS catalog genes that surpass nominal significant cutoff at *p* = 0.05 (grey dotted line) within the categories: (1) major psychiatric disorders; (2) BD-associated traits; and (3) brain-morphometric traits. (**D**) MAGMA gene set associations for each of the predicted miRNA target gene sets, calculated based on summary statistics from Mullins et al. [11]. Grey dotted line marks nominal significant cutoff at *p* = 0.05, while red dotted line marks significance cutoff adjusted for multiple testing.

miRNAs typically serve their gene-regulatory function by binding to complementary sequences in the 3′ untranslated regions of their respective target mRNAs, thereby repressing protein production through translational silencing and by destabilizing the mRNAs (Figure 1) [95]. A computational prediction of 3p and 5p target genes of the selected five putative miRNA biomarkers identified between 36 and 2040 target genes (Figure 4A). The predicted target gene sets that displayed the largest overlap were those of *miR-142-3p* and *-5p*, *miR-106b-5p*, *miR-125a-5p* and *miR-221-3p*—although no gene set shared more than 8% of their genes (Figure 4A). To assess whether the predicted target gene sets shared expression characteristics with their putative governing miRNAs, we tested if their cerebral expression was significantly lower at the time of their respective targeting miRNA’s peak expression. We found that, particularly, the target gene sets of the 5p strands of *miR-106b* and *miR-142* are highly enriched with genes that are downregulated in early childhood—and to some degree in the late fetal stage (Figure 4B). The 3p strands of *miR-142* and *miR-221* similarly showed some degree of enrichment at these stages, whereas the predicted *miR-142-5p* target gene set was slightly enriched with genes that are downregulated at the adolescent stage of development (Figure 4B).

Provided that the identified putative miRNA blood biomarkers are functionally linked to BP pathobiology and/or treatment effect, it is conceivable that their target genes have been implicated with BD pathobiology. Hence, we assessed the significance of the representation of psychiatry-relevant GWAS catalog reported genes in target gene sets. This revealed a significant enrichment of major psychiatric disorder GWS-associated genes in, particularly, the target gene sets of *miR-106b-5p* and *miR-221-3p—*displaying enrichment seen for SZ (Figure 4C). *miR-106b-5p*, *miR-221-3p*, *miR-652-5p,* and *miR-125a-5p* all displayed some degree of enrichment with BD GWAS reported genes (Figure 4C). The same target gene sets further showed a significant enrichment for GWAS reported genes in studies of BD-associated psychometric measures, such as chronotype and risk tolerance (Figure 4C). Intriguingly, for the chronotype and risk tolerance phenotypes, the *miR-142-5p* target gene set showed the overall most significant enrichment, despite showing limited enrichment for BD GWAS reported genes. None of the target gene sets showed strong enrichment for brain imaging GWAS reported genes (Figure 4C).

Genetic variants displaying GWS account for only the most significant, small fraction of total heritability of BD. Hence, to further explore the cumulative genetic BD risk burden in the predicted target gene sets of candidate miRNA biomarkers, we employed a gene set association approach based on the aggregated association of individual genetic markers within the miRNA target gene sets [74]. In line with the observed enrichment of BD GWAS reported genes, the target gene sets of *miR-106b-5p*, *miR-142-5p*, and *miR-125a-5p* all displayed nominal significant gene set association to BD in the assessed GWAS dataset (Figure 4D).

## 4. Discussion

BD is a complex disorder with a strong genetic component. Numerous risk genes and epidemiological risk factors have been identified, and advances has been made toward understanding the biological underpinning of BD. However, patient history and self-reported symptoms remain the basis for both diagnosis and treatment. Due to significant clinical and response heterogeneity in BD, the disorder is frequently misdiagnosed and sub-optimally treated. Identification of peripheral biomarkers that offer patient stratification based on the cumulative risk and/or response profile may thus significantly progress the development of personalized treatment.

miRNAs are embedded in complex regulatory networks, and they play a crucial role in a wide range of biological processes during health and disease. This is highlighted by the estimated >1000 miRNAs that have been associated with pathological conditions [96]. Active secretion of miRNA in vesicles such as exosomes or protein bound plays an important role in endocrine and paracrine regulation of cellular activity, and passive leakage can occur from injured, apoptotic, or necrotic cells [97]. miRNAs produced in most tissues can thus be detected as circulatory miRNAs in bodily fluids such as blood, cerebrospinal fluid (CSF), and tears [98,99]. Circulatory miRNAs are attractive biomarkers because they are often tissue enriched, they can be determined in a quantitative manner, and, most importantly, they are very stable. On a general level, the stability of miRNAs has been estimated to be two to 20-fold that of mRNA and often so at intra-individual stable levels [100]. Accordingly, miRNAs have been proposed as a prognostic tool in oncology with several reaching late-stage clinical trials [101]. In psychiatry, they have additionally been perceived as promising biomarkers because they play a role in post transcriptional gene regulation in many developmental and differentiation processes. Of note, altered miRNA expression has been reported in various brain tissues in patients, manipulation of cerebral miRNA levels has been found to alter behavior in animal studies [58], and miRNAs produced in CNS cells can enter into circulatory bio-fluids.

Importantly, however, environmental factors including toxins and radiation but also adversities, therapeutics, and interventions are known to shape the expression level of miRNAs [102,103,104]. Circulating miRNAs are thus potential biomarkers of both disease status, exposome exposure, and treatment response.

Here we conducted a combined literature and data review that interrogate current experimental evidence supporting the applicability of miRNAs as biomarkers for cumulative risk and response assessment in BD.

Despite the limited number of studies that have investigated the regulation of circulatory miRNAs in BD patients, several miRNAs have been identified that display potential as biomarkers. Particularly, with the inclusion criteria applied in our literature search, we highlighted 12 circulatory miRNAs that have been found to differ in abundancy in studies of blood from BD patients and healthy controls. Although diagnostic criteria are comparable between included studies, differences in study design, study population, medication status, and method of choice, make meta-analyses non-informative. In addition, power issues, the absence of covariates, and a general failure to faithfully estimate the inflation factor in candidate studies may lead to false positives. Yet, several of the reported miRNAs significantly differing between BD cases and controls have previously been associated with other psychiatric disorders or demonstrated to regulate psychiatric disorder susceptibility genes.

Intriguingly, among the 12 putative BD miRNA biomarkers, the expression of five appear to be associated with BD exposome exposure in the form of changes in abundance following treatment or differential expression within BD patients categorized based on their response to treatment.

### 4.1. Asenapine Exposure

Asenapine is a second-generation atypical antipsychotic drug that is approved for the treatment of manic episodes in BD type I patients [105]. In their study, Lim et al. reported that *miR-106b* was significantly more abundant in BD type I patients treated with Asenapine than in corresponding medication-free patients [86]. *miR-106* was also reported upregulated in BD type I patients compared to healthy controls in the study by Camkurt et al. [75]. However, as patients in this study were all manic patients receiving antipsychotic treatment, the reported increase in *miR-106b* abundance in patients compared to controls likely reflects medication status, rather than disease status. Supporting a beneficial effect associated with increased *miR-106b* expression upon antipsychotic treatment in patients, however, we found that *miR-106b* is abundantly expressed in the human brain. Furthermore, the predicted *miR-106b-5p* target gene set is significantly enriched with both SZ and BD GWAS catalog genes, as well as genes associated with related non-clinical phenotypes. In addition, the *miR-106b-5p* target genes displayed robust nominal significant gene-set association with BD. *miR-106b* may thus serve as a response or monitoring biomarker in BD patients receiving antipsychotic treatment—although this will have to be experimentally validated.

### 4.2. Lithium Exposure

Lithium is effective in reducing recurrences, mood switching, and suicide risk, and it is a common first line treatment for BD [106]. As a maintenance treatment, lithium is highly effective in ~1/3 of patients, whereas ~1/3 of patients do not respond to lithium treatment. Although pharmacogenomic studies have failed to establish a statistically significant association between miRNAs and treatment response to lithium in BD in genome-wide analyses [107], expression levels of the putative BD blood biomarkers, *miR-652*, *miR-125a*, *miR-142,* and *miR-221*, all appear to be associated with the lithium response in BD patients.

#### 4.2.1. miR-142

Dysregulation of circulatory *miR-142-3p* was reported in both the study on BD type I patients by Ceylan et al. [84] and in BD type II patients by Lee et al. [59]—although with opposing direction of effect. Specifically, the 3p strand was downregulated in exosomes from BD type I patients on medication, whereas intra- and extracellular *miR-142-3p* was upregulated in medication-free BD type II patients. Of note, *miR-142-3p* was not interrogated in Camkurt et al. but was nominally significantly upregulated in BD type I patient in the study by Fries et al. Differences in endophenotypes and drug administration could explain this discrepancy.

Supporting a role for *miR-142* in shaping the risk profile in BD, the *miR-142* locus displayed multi-marker nominal association with *p*-value < 0.0001 to specifically BD type II. *miR-142* expression consistently peaked in the early childhood across cortical tissues, and in support of a role in post transcriptional gene regulation at this developmental stage, the target gene set of *miR-142-3p* was significantly enriched with genes that are downregulated in the brain exactly in early childhood. Although 5p was the dominant strand in the adult human cortex, both strands were abundant in this tissue. Within brain tissue, *miR-142* expression appeared to be very specific to microglial cells—a cell type that has recently received much attention in BD research [108,109].

While the *miR-142* target gene sets displayed no enrichment of BD GWAS catalog genes, specifically the *miR-142-5p* target gene set showed nominal significant gene set association to BD. Furthermore, the *miR-142-5p* target gene set was very significantly enriched with chronotype-associated genes. Lithium treatment altered the chronotype in BD patients [110], and circadian rhythm dysfunctions have been suggested to predict the clinical response to lithium maintenance treatment [111]. Genetic variation or differential expression of circadian genes have further been associated with lithium response [112,113]. In accordance, *miR-142* expression was increased in lymphoblastoid cells from lithium excellent responders vs. non-responders (both 3p and 5p).

There is thus multilevel evidence that support the applicability of *miR-142* as a predictive and response biomarker in BD.

#### 4.2.2. miR-652

Ceylan et al. reported that *miR-652-3p* was less abundant in exosomes from BD type I patients than in healthy controls [84]. Specifically, its 3p strand, which is the dominant strand in the adult cortex, was also downregulated in lymphoblastoids established from BD type I patients classified as lithium excellent responders. It is thus possible that *miR-652* can be used as a predictive biomarker in the selection of BD type I patients for lithium treatment. Noteworthy, *miR-652* was hardly detected in the adult human brain but rather in the myeloid lineage, and its target gene sets showed no enrichment for psychiatric disorders and traits. A direct link between circulatory *miR-652* dysregulation and the pathobiology of BD was thus not immediately evident. Importantly, in lithium responders, treatment affects the general level of miRNA by DICER mRNA interference [114]. It is, thus, conceivable that circulatory *miR-652* could function as biomarker of lithium administration, as nearly half of the patients investigated by Ceylan et al. were receiving mood stabilizers.

#### 4.2.3. miR-125a

Camkurt et al. report that *miR-125a-3p* was significantly more abundant in blood from BD type I patients than in healthy controls [75], whereas Pisanu et al. found that the, in cortical tissue, much more abundant 5p sister strand was downregulated in lymphoblastoid cells established from BD patients classified as lithium excellent responders [87]. This offers some interesting potential for using *miR-125a* strand abundancies as well as their ratio as a predictive biomarker, although this would have to be established in larger independent samples. Offering some support for a possible role of *miR-125a* in BD pathobiology, *miR-125a* expression peaked in limbic tissues in adolescence and the predicted *miR-125a-5p* target gene set showed nominal gene set association to BD.

#### 4.2.4. miR-221

Finally, Lee et al. reported that *miR-221-5p* was upregulated in BD type II patients vs. healthy controls [59]. Unlike the studies comparing BD type I patients and healthy controls, both the groups in the study by Lee et al. were medication-free—thus more directly supporting the applicability of *miR-221* as a biomarker for health status. Despite being assessed in both the studies by Fries et al. and Ceylon et al., *miR-221* was not found differentially abundant in blood from medicated type I BD patients compared to healthy controls.

Supporting a role for *miR-221* in BD pathobiology, the *miR-221-3p* target gene set was significantly enriched with BD-, chronotype- and risk tolerance GWAS catalog genes. The very specific expression of *miR-221* in excitatory neurons in the human cortex, where it was abundant in the adolescent and adult stage, further suggests a more specialized role for *miR-221* in the brain. Intriguingly, *miR-221* expression appeared to be governed by lithium in blood-derived cells, where treatment increases its expression.

## 5. Conclusions

Based on our comprehensive combined literature and data review, we did not find individual peripheral circulatory miRNAs that convincingly inform on the cumulative risk resulting from heritable factors and exposome exposure in BD. However, we identified several circulatory miRNAs that are differentially abundant in BD patients compared to healthy controls, and which expression is linked to treatment response or to the treatment with antipsychotics or mood stabilizers. Except from *miR-652*, which might have potential as a predictive biomarker regardless, all identified putative biomarkers were widely detected in human brain tissue. In this regard, it is interesting to note that particularly the target genes of *miR-106b*, *miR-125a*, *miR-142* and *miR-221* all displayed gene-set association to BD and/or enrichment of BD GWAS catalog genes. It is thus possible that their dysregulation in blood reflects changes to biological processes in the brain that relates to BD pathobiology or to the effect of treatment.

## Figures and Tables

**Table 1:** Differentially abundant peripheral circulatory miRNAs in bipolar disorder. Combined list of circulating miRNAs with significantly altered abundance in BD patients compared to controls (*p*-value < 0.05) after correction for multiple testing. miRNAs in bold are reported differentially abundant in BD patients in more than one study. SCID-1: Structured clinical interview for DSM-IV-Axis I disorders. SADS-L: Schedule for Affective Disorders and Schizophrenia. BRMS: Bech–Rafaelsen mania scale and the Hamilton depression scale. YMRS: The Young Mania Rating Scale. HAM-D: Hamilton depression scale–17. qRT-PCR: Quantitative Real time-PCR.

miRNA Expression in BD Group Compared to Control Group	Camkurt et al. [75]	Fries et al. [83]	Ceylan et al. [84]	Lee et al. [59]
Setting	Patients recruited from the outpatient psychiatry clinic of Harran University Faculty of Medicine, Turkey.	Subjects were recruited at the Center of Excellence in Mood Disorders at UTHealth, USA.	Manic patients from Shenzhen Kang Ning Hospital, China	Subjects were recruited at the Department of psychiatry, Kaohsiung Veterans General Hospital and National Cheng Kung University Hospital, Taiwan.
Participants	58 medicated BD type I patients and 51 healthy controls	20 medicated BD type I patients and 21 healthy controls.	69 medicated BD type I patients and 41 healthy controls	79 non-medicated BD type II patients and 95 healthy controls.
Diagnostic instrument	SCID-1	SCID-1	SCID-1 YMRS HAM-D	SADS-L
Study material	Extracellular miRNA from peripheral, whole blood.	miRNA from exosomes in peripheral blood	miRNA from exosomes in peripheral blood.	Intra- and extracellular miRNA from peripheral blood, serum.
Method	qRT-PCR	Microarray	qRT-PCR	qRT-PCR
Upregulated	*miR-29a-3p*		*miR-185–5p*	*miR-7-5p*
	*miR-106b-5p*			*miR-23b-3p*
	*miR-107*			***miR-142-3p***
	*miR-125a-3p*			*miR-221-5p*
				*miR-370-3p*
Downregulated			***miR-142-3p***	
			*miR-484*	
			*miR-652-3p*	

**Table 2:** Exposome-associated differentially abundant peripheral circulatory miRNAs in blood/blood cells from BP patients. Combined list of miRNAs with significantly altered abundance in BD patients (*p*-value < 0.05) after correction for multiple testing. Comparisons include treated vs. non-treated; drug-free vs. medicated; responders vs. non-responders; before treatment vs. after treatment. miRNAs in bold are further reported differentially abundant in BD patients vs. healthy controls (Table 1).

	Chen et al. [85]	Lim et al. [86]	Lim et al. [86]	Pisanu et al. [87]
Setting	Recruited from the University of Michigan Depression Center Prechter Bipolar repository, USA.	Recruited from the University Malaya Medical Centre (UMMC) inpatient psychiatric Ward, Malaysia.	Recruited from the University Malaya Medical Centre (UMMC) inpatient psychiatric Ward, Malaysia.	Recruited at the Lithium Clinic of the Clinical Psychopharmacology Centre of the University Hospital of Cagliari, Italy.
Participants	10 BD I patients	5 BD I (manic phase) patients	5 BD I (manic phase) patients	BD patients. 12 Excellent responders and 12 Non-Responders
Diagnostic instrument	Not reported	YMRS	YMRS	SADS-L
Exposure	Lithium (treated vs. non-treated)	Asenapine (drug free vs. medicated)	Risperidone (drug free vs. medicated)	Lithium (responders vs. non-responders)
Study material	Lymphoblastoid cell lines	Whole blood	Whole blood	Lymphoblastoid cell lines
Method	qRT-PCR	Microarray	Microarray	Next Generation Sequencing (NGS)
Upregulated	***miR-221***	*miR-18a-5p*		*miR-148a-3p*
	*miR-152*	*miR-19b-3p*		*miR-22-3p*
	*miR-15a*	*miR-145-5p*		*miR-26b-5p*
		*miR-27a-3p*		*miR-223-3p*
		*miR-148b-3p*		*miR-155-3p*
		*miR-210-3p*		*miR-744-5p*
		*miR-17-3p*		*miR-181a-3p*
		*miR-30b-5p*		*miR-15a-5p*
		***miR-106b-5p***		*miR-148b-3p*
		*miR-339-5p*		*miR-15b-3p*
		*miR-106a-5p*		*miR-454-5p*
		*miR-20a-5p*		*miR-4677-3p*
		*miR-17-5p*		*miR-374a-3p*
		*miR-15a-5p*		*miR-19b-3p*
				*miR-101-3p*
				*miR-148a-5p*
				***miR-142-3p***
				*miR-454-3p*
				***miR-142-5p***
				*let-7f-5p*
				*miR-27a-5p*
				*let-7a-5p*
				*miR-146a-5p*
				*miR-15b-5p*
				*miR-335-3p*
				*miR-421*
				*miR-26a-5p*
Downregulated	*miR-494*	*miR-92b-5p*	*miR-664b-5p*	*miR-320a*
		*miR-1343-5p*	*miR-6778-5p*	***miR-125a-5p***
			*miR-146b-5p*	*miR-574-3p*
				*miR-1273h-3p*
				*miR-9-5p*
				*miR-378a-5p*
				*miR-505-3p*
				*let-7e-5p*
				*miR-138-5p*
				*miR-941*
				***miR-652-3p***
				*miR-130b-3p*
				*miR-345-5p*
				*let-7d-3p*
				*miR-181d-5p*
				*miR-629-5p*
				*miR-574-5p*
				*miR-378a-3p*
				*miR-598-3p*
				*miR-23a-3p*
				*miR-425-5p*
				*miR-197-3p*
				*miR-194-5p*

## Data Availability

Not applicable.

## Supplementary Materials

The following supporting information can be downloaded at: https://www.mdpi.com/article/10.3390/genes13061038/s1, Figure S1: Flowchart outlining the protocol adopted in the search for eligible papers adressing changes in peripheral circulating miRNAs in bipolar disorder; Figure S2: Flowchart outlining the protocol adopted in the search for eligible papers adressing exposome-associated changes in peripheral circulatory miRNAs in bipolar disorder; Figure S3: BD I regional association plots of selected miRNA loci; Figure S4: BD II regional association plots of selected miRNA loci; Table S1: miRNAs located in GWS loci in the largest BD GWAS meta-analysis.
