# Supplementary material for: Circulating miRNAs as Potential Biomarkers for Patient Stratification in Bipolar Disorder: A Combined Review and Data Mining Approach
Source: Genes (Basel). 2022 Jun 10;13(6):1038. doi: 10.3390/genes13061038 (PMC9222282; doi:10.3390/genes13061038)
Supplement: Supplementary file 1 [file genes-13-01038-s001.zip › genes-1702470-supplementary.pdf]

SUPPLEMENTARY MATERIAL

Supplementary Figures:

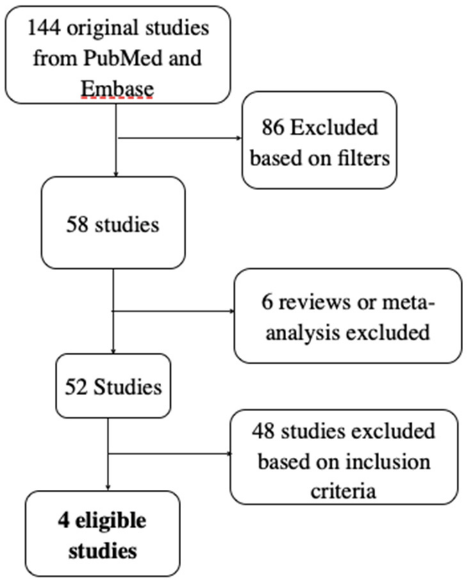

Figure S1: Flowchart outlining the protocol adopted in the search for eligible papers adressing changes in peripheral circulating miRNAs in bipolar disorder. Based on the Preferred Reporting Items for Systematic Reviews and Meta-Analyses (PRISMA) [62].

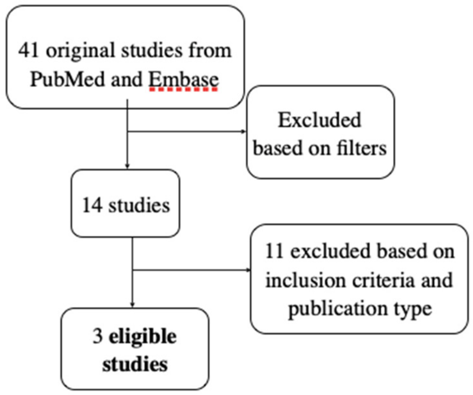

Figure S2: Flowchart outlining the protocol adopted in the search for eligible papers adressing exposome-associated changes in peripheral circulatory miRNAs in bipolar disorder. Based on the Preferred Reporting Items for Systematic Reviews and Meta-Analyses (PRISMA) [62].

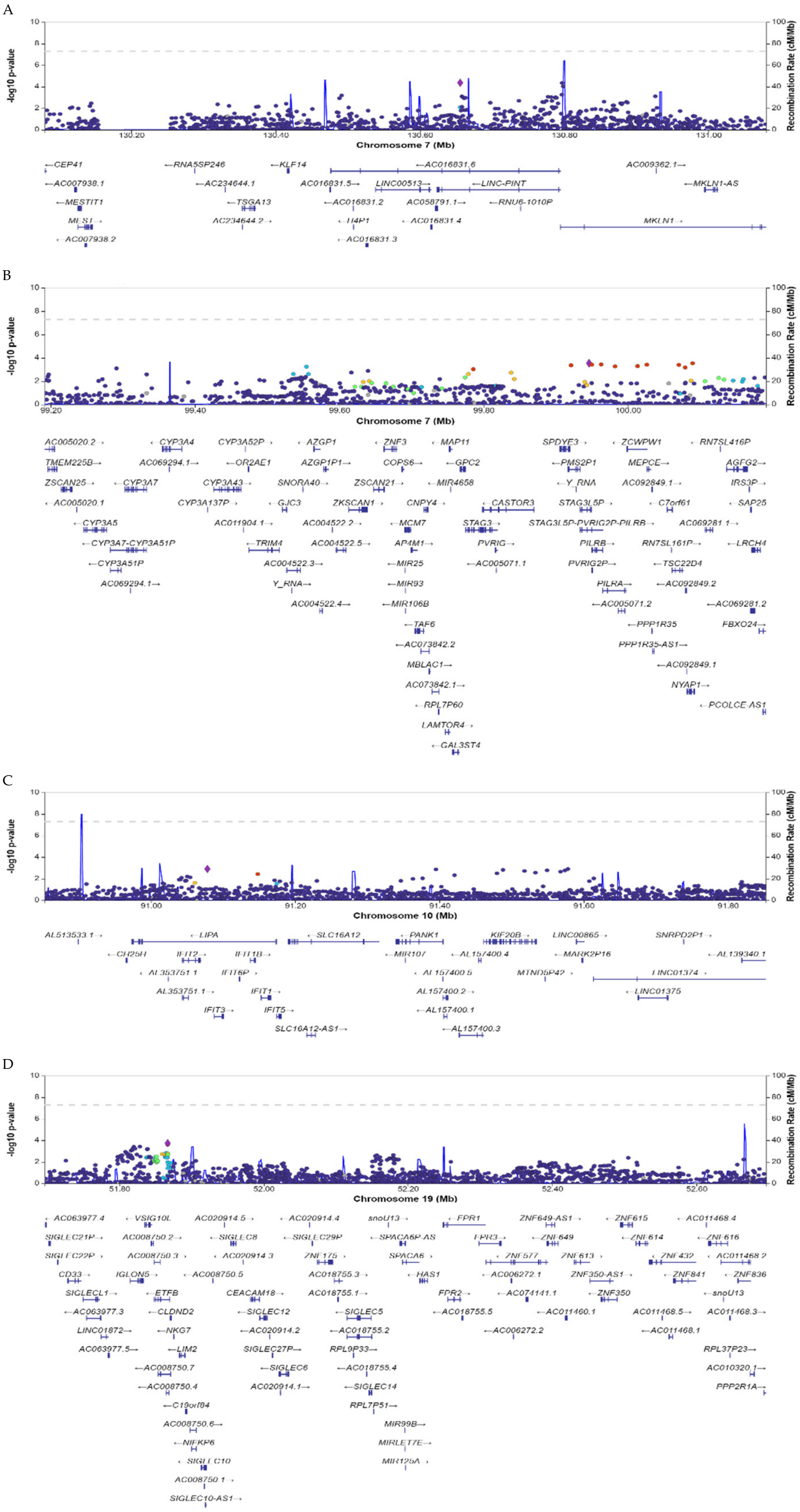
A
-log10 p-value
Recombination Rate (cM/Mb)
Chromosome 7 (Mb)
B
-log10 p-value
Recombination Rate (cM/Mb)
Chromosome 7 (Mb)
C
-log10 p-value
Recombination Rate (cM/Mb)
Chromosome 10 (Mb)
D
-log10 p-value
Recombination Rate (cM/Mb)
Chromosome 19 (Mb)

E

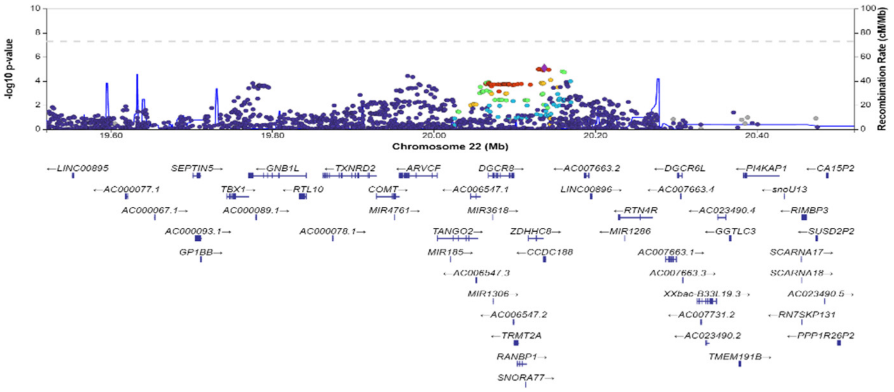
-log10 p-value
Recombination Rate (cM/Mb)
Chromosome 22 (Mb)

F

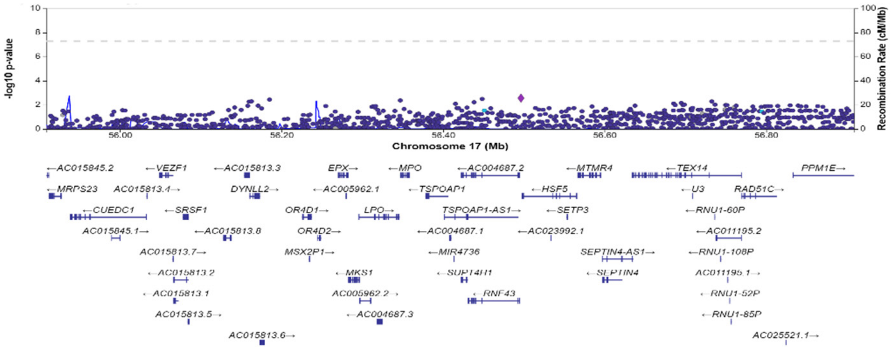
-log10 p-value
Recombination Rate (cM/Mb)
Chromosome 17 (Mb)

G

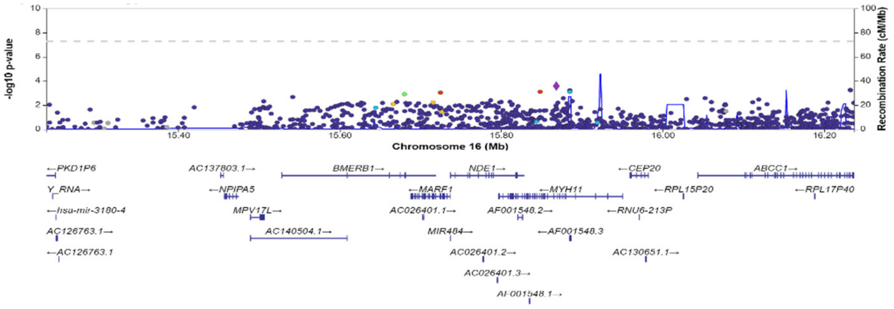
-log10 p-value
Recombination Rate (cM/Mb)
Chromosome 16 (Mb)

H

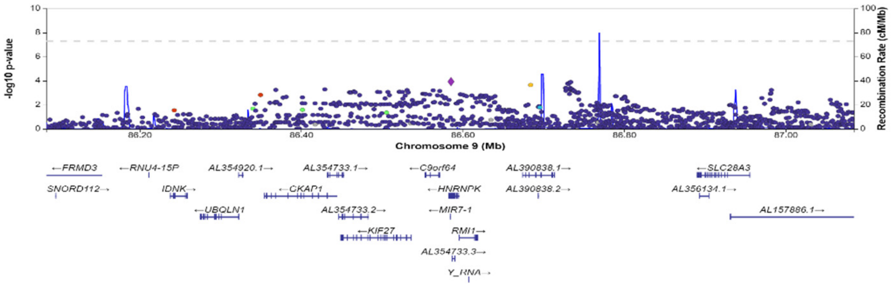
-log10 p-value
Recombination Rate (cM/Mb)
Chromosome 9 (Mb)

I

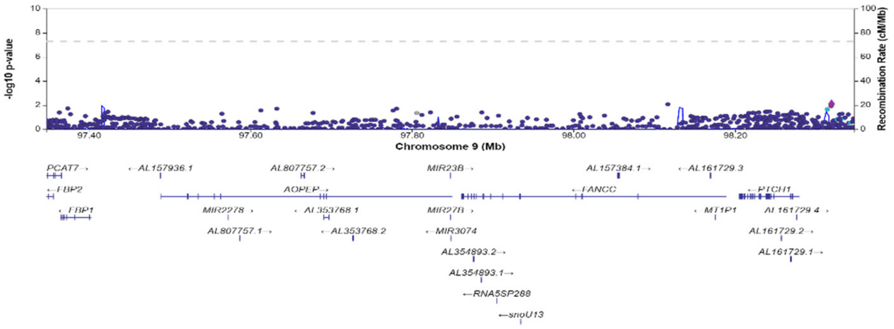
-log10 p-value
Recombination Rate (cM/Mb)
Chromosome 9 (Mb)

J

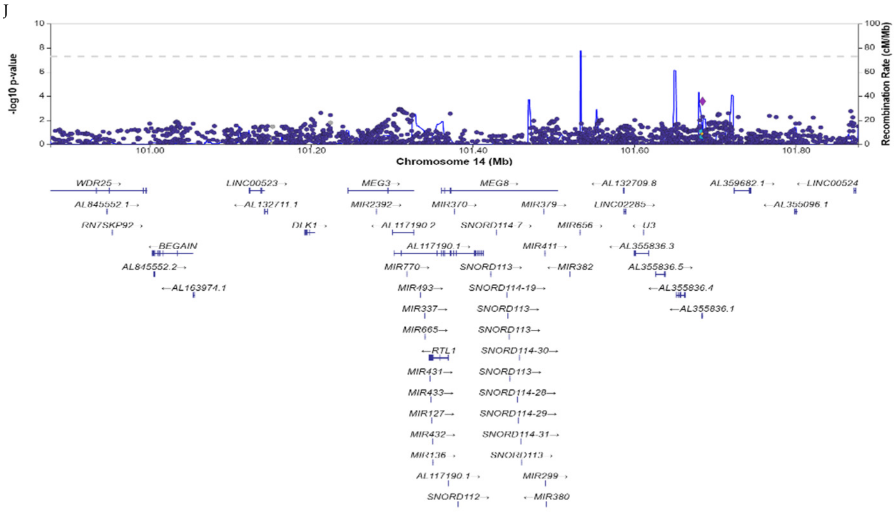

Figure S3: BD I regional association plots of selected miRNA loci. A) *mir-29a* B) *mir-106b* C) *mir-107* D) *mir-125a* E) *mir-185* F) *mir-142* G) *mir-484* H) *mir-7* I) *mir-23b* J) *mir-370*. *mir-652* and *mir-221* are both located on the X chromosome that is not covered by the summary statistics. The purple diamond indicates the most significant single-nucleotide polymorphism (SNP) of each region, and nearby SNPs are color-coded to show their lD relationships with the top SNP (r2<0.2; dark blue, 0.2≤r2<0.4; light blue, 0.4≤r2<0.8; green, r2>0.8; red) based on Hapmap3 EUR.

A

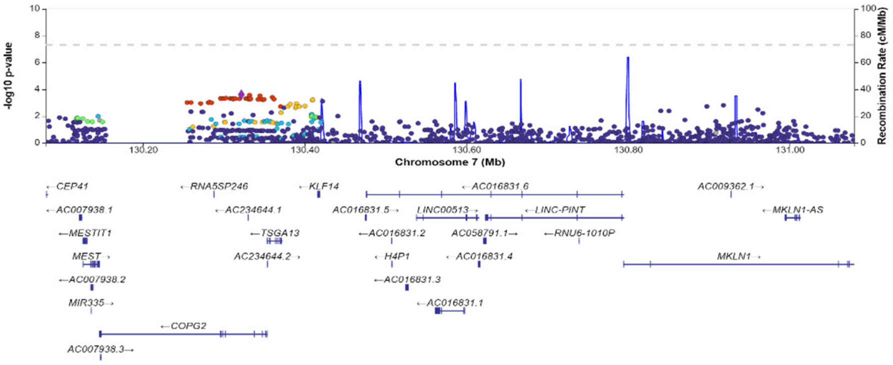

B

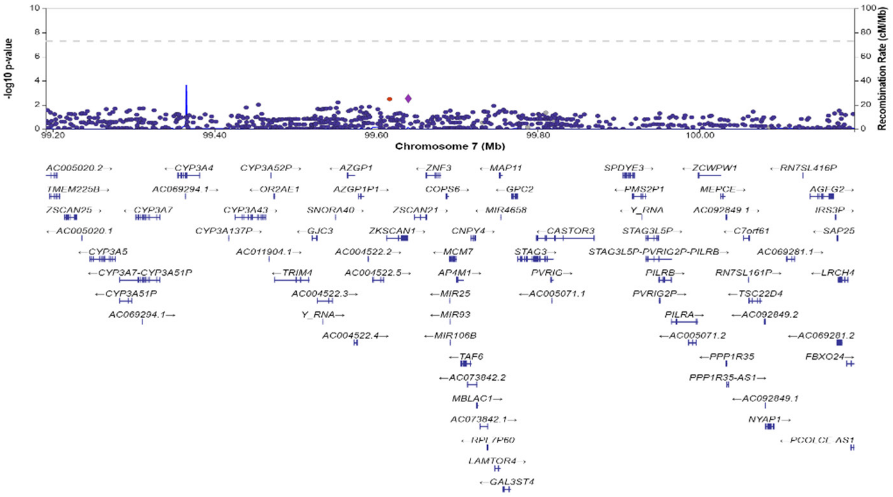

C

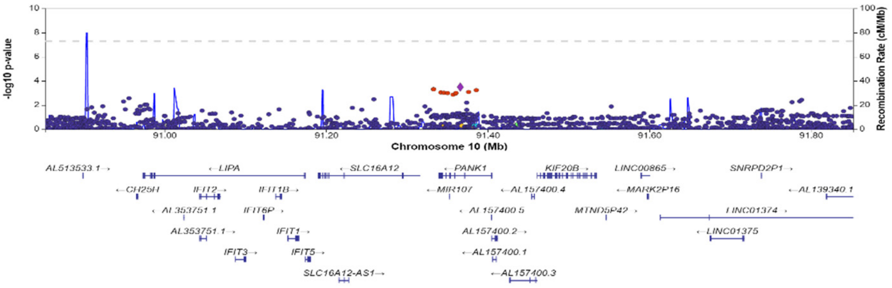

-log10 p-value
Recombination Rate (cM/Mb)
Chromosome 10 (Mb)
91.00
91.20
91.40
91.60
91.80
AL513533.1
LIPA
SLC16A12
PANK1
KIF20B
LINC00865
SNRPD2P1
CH25H
IFIT2
IFIT1B
MIR107
AL157400.4
MARK2P16
AL139340.1
AL353751.1
IFIT6P
AL157400.5
MTND5P42
LINC01374
IFIT1
AL157400.2
LINC01375
IFIT3
IFIT5
AL157400.1
SLC16A12-AS1
AL157400.3

D

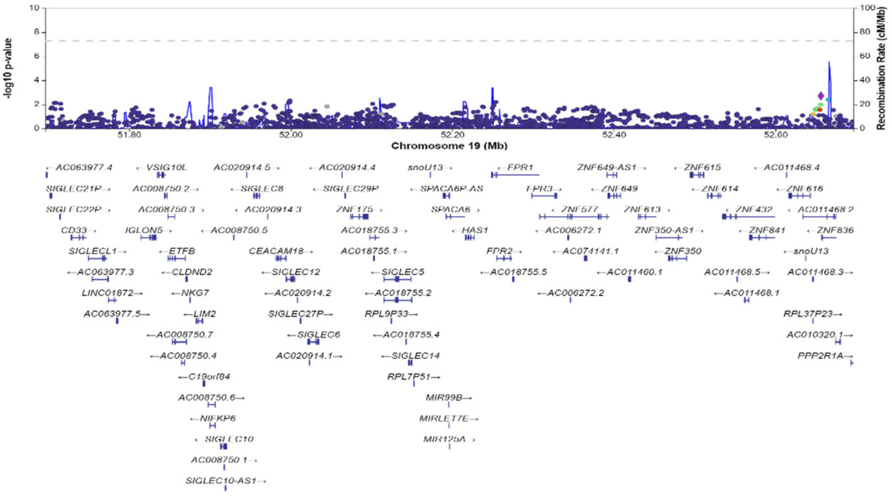

-log10 p-value
Recombination Rate (cM/Mb)
Chromosome 19 (Mb)
51.80
52.00
52.20
52.40
52.60

E

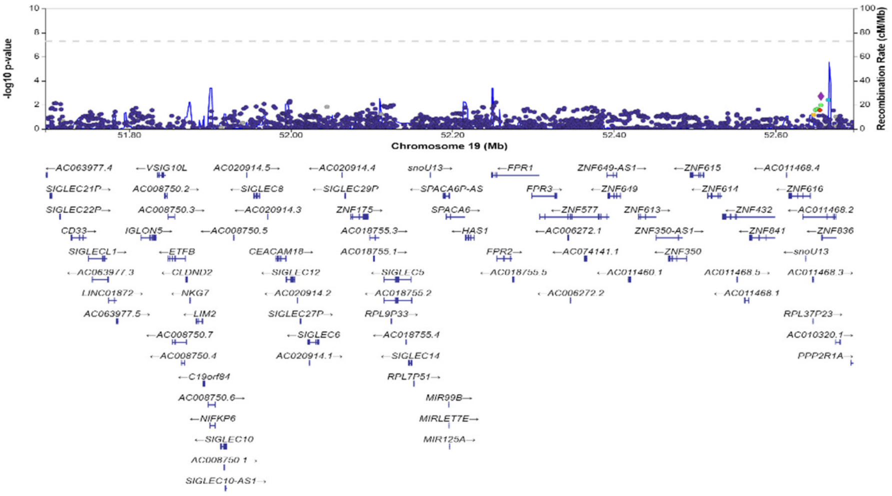

-log10 p-value
Recombination Rate (cM/Mb)
Chromosome 19 (Mb)
51.80
52.00
52.20
52.40
52.60

F

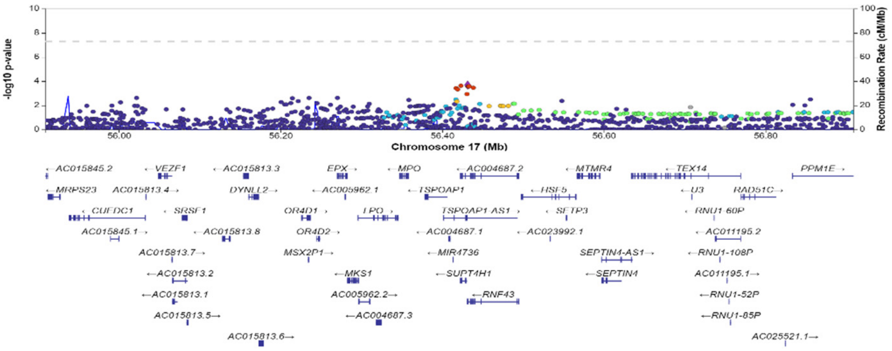

-log10 p-value
Recombination Rate (cM/Mb)
Chromosome 17 (Mb)
56.00
56.20
56.40
56.60
56.80

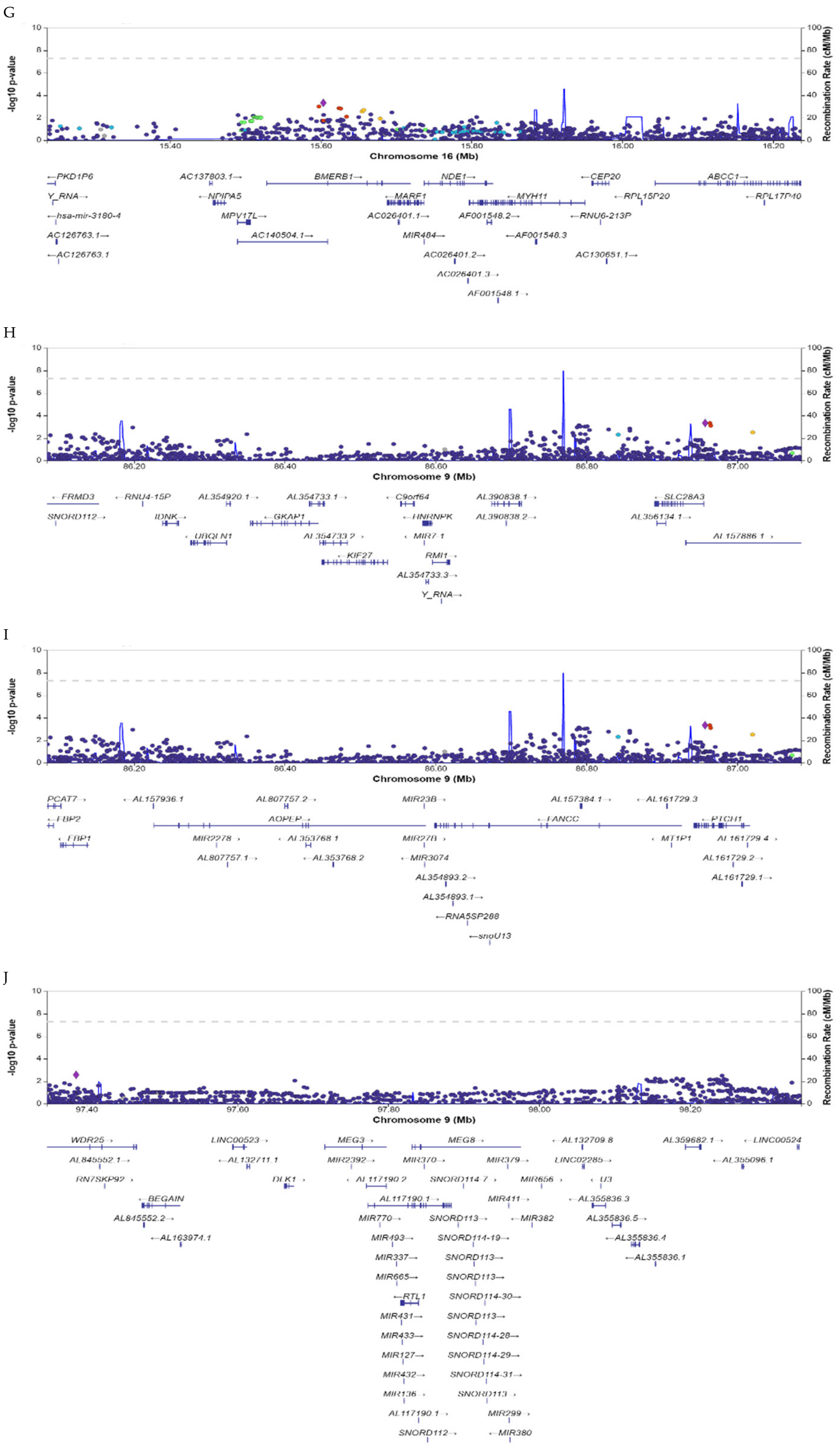

Figure S4: BD ii regional association plots of selected miRNA loci. **A**) *mir-29a* **B**) *mir-106b* C) *mir-107* D) *mir-125a* E) *mir-185* F) *mir-142* G) *mir-484* H) *mir-7* I) *mir-23b* J) *mir-370*. *mir-652* and *mir-221* are both located on the X chromosome that is not covered by the summary statistics. the purple diamond indicates the most significant single-nucleotide polymorphism (SNP) of each region, and nearby SNPs are color-coded to show their LD relationships with the top SNP ($r2<0.2$; dark blue, $0.2\leq r2<0.4$; light blue, $0.4\leq r2<0.8$; green, $r2>0.8$; red) based on Hapmap3 EUR.

Supplementary Table:

Table S1. | miRNAs located in GWS loci in the largest BD GWAS meta-analysis.

miRNA	GWAS locus	lead SNP (rsID)	Chromosome	Position	p-value	Distance from lead SNP (kb)
miR-3127	3	rs4619651	2	96750416	4,78E-11	-47,862
miR-4774	5	rs13417268	2	168625327	2,05E-08	42,384
miR-8064	9	rs2336147	3	52592427	3,62E-13	298,119
miR-LET7G	9	rs2336147	3	52592427	3,62E-13	324,066
miR-135A1	9	rs2336147	3	52592427	3,62E-13	-254,125
miR-2113	18	rs13195402	6	26463347	5,75E-15	-684,279
miR-3143	19	rs1487445	6	98117335	1,48E-15	92,804
miR-1183	22	rs12668848	7	1981360	1,9E-09	137,18
miR-4655	24	rs6954854	7	21452971	5,94E-10	-18,175
miR-124-1	29	rs62489493	8	9906321	2,64E-11	2,849
miR-661	31	rs6992333	8	143919209	1,62E-09	-25,982
miR-708	38	rs174592	11	61851136	9,92E-14	35,896
miR-5579	38	rs174592	11	61851136	9,92E-14	58,575
miR-611	42	rs12289486	11	79381483	3,3E-08	-40,743
miR-1908	42	rs12289486	11	79381483	3,3E-08	-20,626
miR-627	47	rs4447398	15	42612706	2,61E-09	413,04
miR-4515	48	rs62011709	15	82863022	1,43E-08	-204,313
miR-6884	55	rs61554907	17	40064179	1,64E-08	37,77
miR-6782	57	rs67712855	20	45053910	4,22E-11	846,071
miR-4766	60	rs5758064	22	40757875	2,01E-08	-56,083
